# Screening the toxicity profile and genotoxicity mechanism of excess manganese confirmed by spectral shift

**DOI:** 10.1038/s41598-022-25657-6

**Published:** 2022-12-05

**Authors:** Cihat Tümer, Kültiğin Çavuşoğlu, Emine Yalçin

**Affiliations:** 1 Department of Biology, Institute of Science, Giresun University, Giresun, Turkey; 2 Department of Biology, Faculty of Arts and Sciences, Giresun University, 28200 Giresun, Turkey

**Keywords:** Biochemistry, Cell biology, Genetics

## Abstract

In this study, the toxicity induced by excessive doses of manganese (MnCl_2_), which is one of the essential trace elements for the continuation of the metabolic activities of the organisms, was investigated with the help of the *Allium* test. Toxicity was investigated by using physiological (percent germination, root length, weight gain), cytogenetic [mitotic index (MI), micronucleus (MN), chromosomal abnormalities (CAs)], biochemical [malondialdehyde (MDA), superoxide dismutase (SOD) catalase (CAT)] and anatomical (root tip meristematic cell damage) parameters. *Allium cepa* L. bulbs were divided into four groups as one control and three treatments. The control group was germinated with tap water, and the treatment groups were germinated with 250, 500 and 1000 µM doses of MnCl_2_. The germination process was continued for 72 h without interruption. At the end of the period, the root tips were collected, washed in distilled water and made ready for microscopic and spectrophotometric analyzes with the help of routine preparation techniques. As a result, the highest germination percentage, root length, weight gain and MI, and the lowest MN frequency, CAs numbers, MDA level, SOD and CAT enzyme activities were determined in the control group (group I). MnCl_2_ exposure caused a decrease in physiological parameter values and an increase in cytogenetic (except MI) and biochemical parameter values, depending on the dose. MnCl_2_ exposure induced MN and CAs such as fragment, sticky chromosome, vagrant chromosome, unequal distribution of chromatin and bridge. This genotoxic effect of MnCl_2_ was associated with DNA–MnCl_2_ interaction, and this interaction was also confirmed by bathochromic and hypochromic shifts in spectral analysis. Anatomical damages such as epidermis cell damage, flattened cell nucleus, cortex cell damage and cortex cell wall thickening were observed after MnCl_2_ treatment. As a result, it has been determined that excessive doses of the trace element Mn cause physiological, cytogenetic, biochemical and anatomical toxicity and *A. cepa* test material is a reliable bio-indicator in determining this toxicity.

## Introduction

The elements that are present in very small amounts in the organism but are absolutely necessary for the cells to perform their functions at the biological, chemical and molecular level are called trace elements. They are also known as micro or minor elements. These elements act as cofactors of many enzymes responsible for chemical events, especially at the cellular level. They also serve as atomic centers to stabilize enzyme and protein structures. The main trace elements found in organisms are boron (B), chromium (Cr), cobalt (Co), copper (Cu), fluorine (F), iodine (I), iron (Fe), molybdenum (Mo), selenium (Se), silicon (Si), vanadium (V), zinc (Zn) and manganese (Mn). Trace elements have vital importance in plants. They are involved in plant growth, enzyme production, protein synthesis and hormone regulation. Very small amounts of trace elements such as Fe, Cl, Zn, Ni, Cu, B, Co, Mo and Mn are required to perform all these tasks. Excessive increases in the concentrations of these elements can change the physicochemical and biological properties of the soil and cause various negative effects on the plants growing in these soils. Although trace elements are found naturally in the soil, their amounts in the soil are increasing day by day as a result of various activities such as industrial wastes, burning fossil fuels, irrigation with dirty water, excessive fertilization and pesticide pollution containing heavy metals^1^.

Manganese (Mn) is one of the trace elements with heavy metal properties. It is a pinkish gray element with the symbol Mn. It is located in group 7 of the periodic table and its atomic number is 25. Its boiling point is 2061 °C, its melting point is 1247 °C and its density is 7.44 g/cm^3^. Mn is an element commonly found in the earth's crust. Therefore, it is the 12th most abundant element on earth. Mn is not naturally found in pure form. Oxides, carbonates and silicates are the most important Mn-containing minerals. Mn is found in many iron ores, coal and crude oil. Mn is used as a desulfurizing additive and alloy component in metallurgical processes. It is also used in battery production, chemical and glass manufacturing, leather and textile industry and as fertilizer. Organic carbonyl compounds of Mn are used as fuel additives, smoke inhibitors and anti-knock additives in gasoline. Australia, Brazil, South Africa, Gabon and India are the main Mn producing countries. Mn is concentrated in the bones, kidneys, liver and pancreas in humans. It plays a role in many processes such as bone formation (calcium absorption) and development, healing of wounds (blood clotting), synthesis of proteins, hormone functions, regulation of blood sugar and fulfillment of immune functions. Mn is abundant in many vegetables and fruits such as nuts, walnuts, almonds, pumpkin seeds, grapes, avocados, pineapple, peas, spinach, beans, blackberries, broccoli, asparagus, cereals, eggs and oranges. In addition, mineral waters also contain large amounts of Mn. In humans, Mn deficiency can cause fatigue, weight loss, forgetfulness, irritability, and learning and understanding difficulties. In addition, Mn deficiency can cause problems in the digestive system, brain and nervous system, muscles, kidneys, heart and vessels. However, overexposure to Mn doses can cause neuro-chemical, neuro-behavioral and neuro-endocrine changes^2,3^.

Mn is an essential microelement for organisms. Mn found in plants plays a role as a cofactor in the activation of about 35 enzymes such as SOD, CAT, pyruvate carboxylase and phospho-enolpyruvate carboxykinase. It is involved in ATP synthesis, RuBP carboxylase reactions, and biosynthesis of fatty acids, acyl lipids and proteins. Mn is also required for the biosynthesis of chlorophyll, aromatic amino acids, lignin and flavonoids. It participates in the structure of proteins and enzymes that play a role in photosynthesis. Its deficiency affects the water separation system of photosystem II, which provides electrons for photosynthesis in chloroplasts. Its excess is thought to damage the photosynthetic system. It has been suggested that increasing Mn concentrations may inhibit the biosynthesis of chlorophyll and carotenoids, causing a decrease in the photosynthetic electron transport rate and thus a slowdown in photosynthesis. It has also been reported that high Mn concentrations can cause root and shoot shortening. Acidic soils are ideal for Mn toxicity. As the pH ratio decreases, the Mn^2+^ form increases in the soil solution. This form of Mn is rapidly taken up by plant roots and can be easily transported to cells. Excessive Mn levels in plant tissues can cause oxidative stress by changing enzyme activity and the uptake processes of water and mineral substances such as Ca, Mg, Fe and P. Mn as a SOD cofactor, it participates in the defense of the plant against oxidative stress produced by active oxygen and free radicals, which are very dangerous for plants. Mn-SOD also increases the tolerance of plant cells to environmental stresses such as salt stress. Low amounts of Mn are absolutely necessary for the normal nutrition and development of plants. However, excessive amounts are extremely toxic to plants. Excessive amounts of Mn can cause a decrease in growth rate in plants, chlorosis and necrosis in leaves^4^.

The effects of Mn in plants are mostly focused on the negative effects of Mn deficiency. However, the number of studies on the negative effects of excessive Mn exposure is very limited. On the other hand, the number of studies on the negative effects of excessive Mn exposure in *Allium* species, which is a cosmopolitan cultivar and widely consumed all over the world, is very limited. Therefore, there is a need for an increase in the number of such studies. Besides, agricultural activities are of great importance for the maintenance of life. For this reason, the use of fertilizers containing trace elements is increasing day by day in order to increase soil and plant productivity all over the world. This situation causes an increase in the amount of trace elements in the soil at an undesirable level, and can cause negative effects by entering the structure of many organisms, especially the agricultural products produced in these elements and the human being fed from these products. The aim of this study is to investigate the multifaceted toxicity induced by excessive MnCl_2_ exposure in *A. cepa* with the help of physiological, cytogenetic, biochemical and anatomical parameters.

## Materials and methods

### Test material and chemical

*Allium cepa* bulbs (2n = 16) were purchased from a commercial market in Giresun province, and Manganese (II) chloride (MnCI_2_) was purchased from Merck (CAS No: 7773-015).

### Dose preference

MnCI_2_ doses of 250, 500 and 1000 µM were determined considering the toxic dose ranges recommended for different plant species by Millaleo et al.^4^.

### Principles of group formation and experimental practice

**Figure 1:**
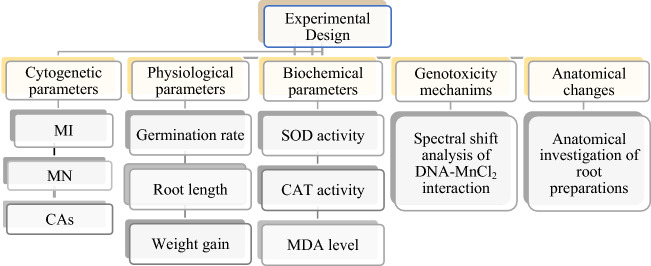
Experimental stages of study. *MI* mitotic index, *MN* micronucleus, *CAs* chromosomal abnormalities, *SOD* superoxide dismutase, *CAT* catalase, *MDA* malondialdehyde.

A total of 4 groups were formed from healthy and almost equal-sized *A. cepa* bulbs as 1 control and 3 applications. Bulbs were placed in glass beakers. The bulbs of the control group were germinated with tap water, and the bulbs of the treatment group were germinated with three different doses of MnCl_2_ (250, 500 and 1000 µM). The germination process was continued for 72 h at room temperature without interruption. Experimental sets were checked at 24 h intervals, and the missing solutions were added. At the end of the period, the germinated root tips were washed with distilled water and made ready for experimental measurements and microscopic examinations by applying routine homogenization and crushing preparation techniques. Experimental research on plant samples, including the supply of plant material, complies with institutional, national and international guidelines and legislation. All parameters investigated throughout the study are summarized in Fig. 1.

### Physiological parameter measurements

The effect of MnCl_2_ on root elongation was determined on the basis of radicle formation, which is the primary structure in the embryo that provides root formation. The root lengths were determined by measuring the radicle lengths of the bulbs with the help of a millimetric ruler. The effect of MnCl_2_ on the weight was calculated by weighing the bulb weights with precision balance before and after the experimental application, and the effect on germination was calculated with the help of the following Eq. (1)^5^.

1 $$ {\text{Germination }}\left( \% \right) \, = \, \left[ {{\text{number}}\,{\text{of}}\,{\text{germinated}}\,{\text{seeds}}} \right]/\left[ {{\text{total}}\,{\text{number}}\,{\text{of}}\,{\text{seeds}}} \right] \, \times { 1}00. $$

### Cytogenetic parameter observations

The acetocarmine crushing technique was used to observation CAs and MN. Fresh root tips were cut about 1 cm long. It was fixed in the “Clarke” fixator for 2 h. It was washed in ethyl alcohol (96%) for 15 min, hydrolyzed in 1 N HCl for 17 min at 60 °C and incubated in glacial acetic acid (45%) for 30 min. As a final process, the root tips were stained in acetocarmine for 24 h, taken on a slide, crushed by pressing the coverslip and examined under the Irmeco IM-450 TI model research microscope and photographed at 400× magnification^6^. MN determination was made based on the following three criteria determined by Fenech et al.^7^. These:

- MN should be about 1/3 of the cell nucleus,
- MN should be stained the same color as the cell nucleus,
- when the MN and the cell nucleus come into contact, the boundary between them should be clearly distinguishable.

CAs and MN counts were repeated twice (by two different observers) to increase the percentage of accuracy.

MI, which shows the ratio of cells undergoing mitosis to all cells in the meristematic cell population, was calculated using the Eq. (2) below.

2 $$ {\text{MI }}\left( \% \right) \, = \, \left[ {{\text{number}}\,{\text{of}}\,{\text{cells}}\,{\text{undergoing}}\,{\text{mitosis}}} \right]/\left[ {{\text{total}}\,{\text{number}}\,{\text{of}}\,{\text{cells}}} \right] \, \times { 1}00. $$

### Spectral measurements for DNA–MnCl_2_ interaction

To elucidate the genotoxicity mechanism of manganese chloride, the UV spectrum of the DNA and DNA–MnCl_2_ complex were investigated. For this purpose, DNA was isolated from *A. cepa* root tip cells and then used for spectral measurements. DNA isolation from *Allium* root samples was carried out according to the method suggested by Macar et al.^8^. DNA–MnCl_2_ interaction was evaluated by investigating the change in absorbance of DNA and DNA–MnCl_2_ mixtures (1:1, 1:2, 1:3). The UV absorption spectrum was obtained in the range of 240–280 nm. UV absorption spectra were recorded on the Mapada UV-6100PCS double beam spectrophotometers.

### Biochemical parameter analyzes

#### Measurement of MDA levels

MDA levels were measured using the method suggested by Unyayar et al.^9^. For each group, 0.5 g of fresh root tips was homogenized in 1 mL of trichloroacetic acid (5%). The homogenate was transferred to a new tube and centrifuged at 12,000*g* for 10 min. The supernatant and thiobarbituric acid (0.5%) were transferred to a new tube in equal volumes and incubated in trichloroacetic acid solution (20%) for 30 min at 96 °C. Following incubation, the tubes were rapidly cooled and centrifuged at 10,000*g* for 5 min. The absorbance of the supernatant was recorded at 532 nm and the MDA level was shown as μM/g FW.

#### Measurement of enzyme activities

Enzyme extraction and preparation were carried out at + 4 °C. 0.5 g of fresh root tips were washed with distilled water and homogenized with a mortar in 5 mL of cold sodium phosphate buffer (50 mM, pH 7.8). The homogenates were centrifuged at 10,500*g* for 20 min and the supernatants were stored at + 4 °C until analysis^10^.

### SOD measurement

SOD activity was measured by applying the method suggested by Beauchamp and Fridovich^11^. A total of 3 mL of reaction solution was prepared [1.5 mL 0.05 M sodium phosphate buffer (pH 7.8), 0.3 mL 750 µM nitroblue tetrazolium chloride, 0.3 mL 130 mM methionine, 0.3 mL 20 µM riboflavin, 0.3 mL 0.1 mM EDTA-Na_2_, 0.01 mL 4% (w/v) insoluble polyvinylpyrrolidone, 0.01 mL enzyme extract and 0.28 mL de-ionized water]. The reaction was started by placing the tubes containing the solution under two 15 W fluorescent lamps for 10 min and ended by keeping the tubes in the dark for 10 min. The absorbance was read at 560 nm and recorded. One unit of SOD activity was defined as the amount of SOD required to produce 50% inhibition of nitroblue tetrazolium chloride reduction under experimental conditions, and the SOD activity was shown as U/mg FW^10^.

### CAT measurement

CAT activity was measured by applying the method suggested by Beers and Sizer^12^. CAT activity was determined with the help of UV–Vis spectrophotometry in a total of 2.8 mL reaction mixture [1.5 mL 200 mM monosodium phosphate buffer (pH 7.8), 1.0 mL distilled and 0.3 mL 0.1 M hydrogen peroxide (H_2_O_2_)] at 25 °C. The reaction was initiated by adding 0.2 mL of enzyme extract. CAT activity was measured by monitoring the decrease in absorbance at 240 nm as a result of H_2_O_2_ consumption. CAT activity is expressed as units per minute per root tip fresh weight and is shown as OD_240nm_ min/g^10^.

### Anatomical observations

Root tips were cut about 0.5 cm long, washed with distilled water, placed between styrofoam and cross-sectioned using a sterile razor blade. The sections taken were placed on the slide, stained with methylene blue (5%) for 2 min, covered with a coverslip and examined under the Irmeco IM-450 TI model research microscope and photographed at 200× magnification^13^.

### Statistical analyzes

Statistical analysis of the data was made with the help of SPSS Statistics 22 (IBM SPSS, Turkiye) package program. Data are shown as mean ± standard deviation (SD). Statistical significance between the data was determined using one-way analysis of variance, “One-way Anova” and “Duncan” tests. It was considered statistically significant when the p value was less than 0.05.

## Results and discussion

### Physiological observations

The effect of MnCl_2_ exposure on the investigated physiological parameters is shown in Table 1. The highest germination percentage, root length and weight gain were determined in the control group (group I). In this group, germination rate of 100%, average 7.60 cm root length and average 5.96 g weight gain were determined. MnCl_2_ exposure caused a statistically significant (p < 0.05) decrease in the values of all three physiological parameters examined, depending on the dose. The greatest decrease was measured in Group IV, which was exposed to 1000 µM dose of MnCl_2_. Compared to the control group (group I), germination decreased by 28%, root length approximately 2.4 times and weight approximately 3.0 times in Group IV.

**Table 1:** Physiological effects of MnCl_2_ toxicity.

Groups	Germination percentage (%)	Root length (cm)	Initial weight (g)	Final weight (g)	Weight gain (g)
Group I	100	7.60 ± 0.98^a^	13.78 ± 1.54	19.74 ± 1.86	+ 5.96^a^
Group II	91	6.40 ± 0.92^b^	13.50 ± 1.51	18.25 ± 1.80	+ 4.75^b^
Group III	84	5.00 ± 0.83^c^	13.62 ± 1.50	17.10 ± 1.74	+ 3.48^c^
Group IV	72	3.20 ± 0.68^d^	13.80 ± 1.56	15.78 ± 1.66	+ 1.98^d^

Group I: Control, Group II: 250 µM MnCl_2_, Group III: 500 µM MnCl_2_, Group IV: 1000 µM MnCl_2_. Data are shown as mean ± SD. 50 bulbs were used to determine germination percentage, and 10 bulbs were used to determine root length and weight gain. The averages shown with different letters^(a–d)^ in the same column are statistically significant at p < 0.05.

These results are similar to the results of a limited number of studies on the physiological toxicity induced by excessive Mn exposure in plants. For example; Zhao et al.^14^ reported that Mn exposure at 2, 4, 6, 8, 10, and 12 mM doses decreased primary root growth in *Arabidopsis*, depending on the application dose. Rai et al.^15^ observed that 50, 100, 200, 300, 500 and 1000 ppm Mn doses decreased the germination rate and caused inhibition in shoot and root growth depending on the concentration increase in *Vigna radiata* L. seeds, a type of pod.

Our study suggests that the decrease observed in physiological parameters as a result of MnCl_2_ exposure may be due to the fact that Mn reduces the uptake of micro and macro elements and mitosis cell division of plant roots. Because Joardar Mukhopadhyay and Sharma^16^ reported that excess Mn inhibits the uptake, transport and utilization of many essential elements such as Ca, Fe, Cu, Al, Si, Mg, K, P and N by plant roots. Zhao et al.^14^ determined that Mn toxicity inhibits root elongation by reducing the division potential of meristematic cells at the root tips.

### Cytogenetic observations

**Figure 2:**
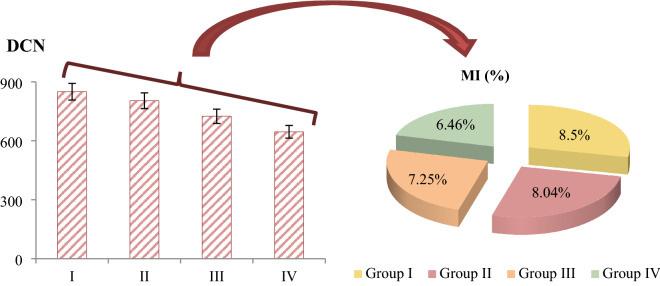
The effects of MnCl_2_ on dividing cell number (DCN) and MI (%).Group I: Control, Group II: 250 µM MnCl_2_, Group III: 500 µM MnCl_2_, Group IV: 1000 µM MnCl_2_.

**Figure 3:**
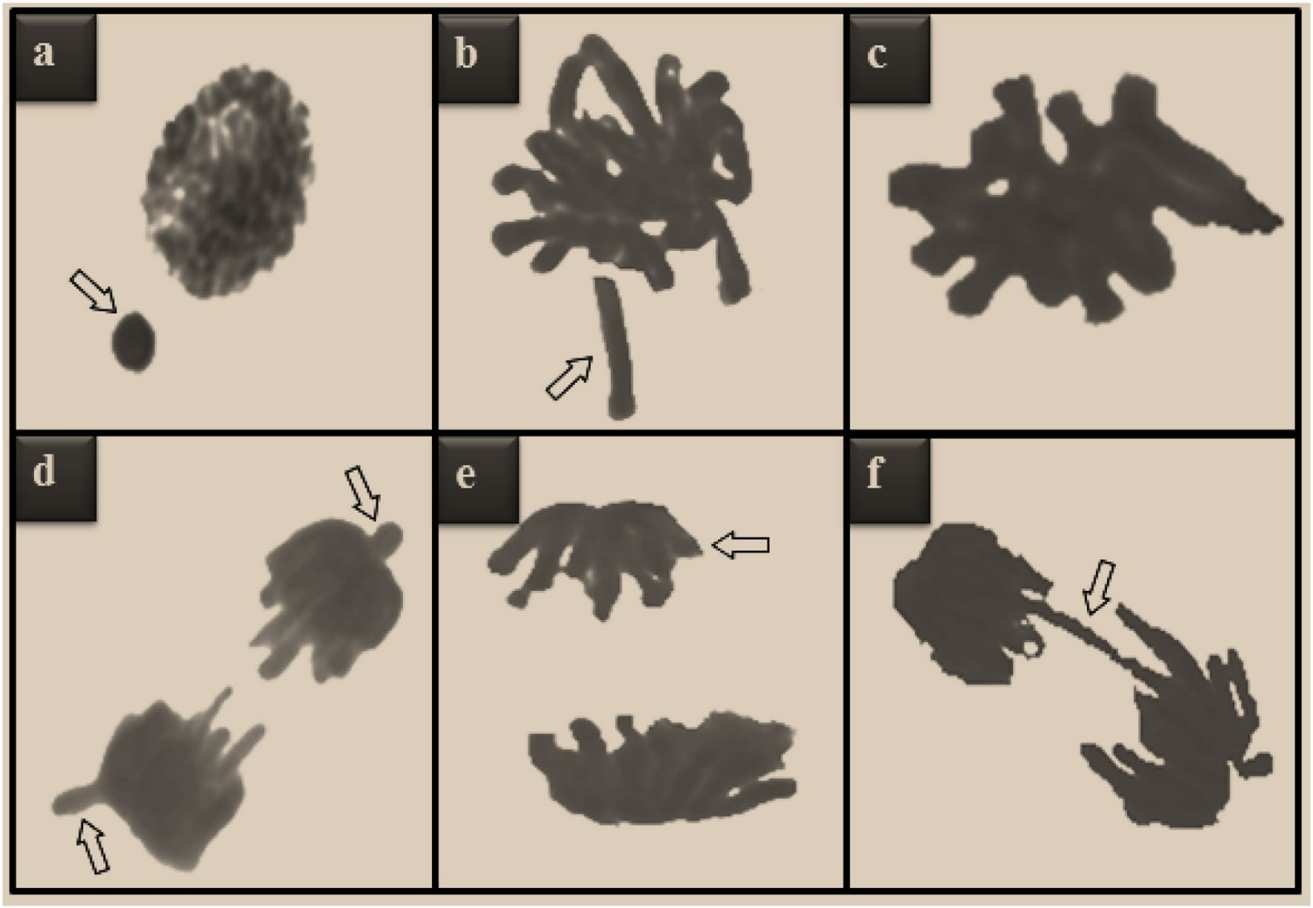
CAs types induced by MnCl_2_ toxicity. MN (**a**), fragment (**b**), sticky chromosome (**c**), vagrant chromosome (**d**), unequal distribution of chromatin (**e**), bridge (**f**).

Cytogenetic abnormalities induced by MnCl_2_ exposure in root tip meristematic cells are shown in Table 2, Figs. 2 and 3. The highest MI value, lowest MN and CAs frequencies were detected in the control group (group I). In this group, 8.50% MI and negligible number of MN were detected. In addition, no CAs was observed in this group, except for a few sticky chromosome and unequal distribution of chromatin. MnCl_2_ exposure caused a statistically significant (p < 0.05) decrease in MI and a statistically significant (p < 0.05) increase in the frequencies of MN and CAs, depending on the dose. MnCl_2_ exposure induced CAs such as fragment, sticky chromosome, vagrant chromosome, unequal distribution of chromatin and bridge in root tip meristem cells. The greatest effect of MnCl_2_ on chromosomes was fragment formation. Compared to the control group (group I), MI decreased by approximately 2% and MN increased approximately 379 times in Group IV exposed to 1000 µM dose of MnCl_2_.

**Table 2:** MN and CAs frequencies induced by MnCl_2_.

Abnormalities	Group I	Group II	Group III	Group IV
MN	0.16 ± 0.22^d^	20.5 ± 1.34^c^	33.4 ± 2.98^b^	60.6 ± 5.48^a^
FRG	0.00 ± 0.00^d^	13.8 ± 1.12^c^	27.9 ± 2.75^b^	50.2 ± 4.83^a^
SC	0.12 ± 0.17^d^	12.4 ± 1.05^c^	19.6 ± 1.40^b^	38.7 ± 3.74^a^
VC	0.00 ± 0.00^d^	10.6 ± 0.97^c^	19.5 ± 1.38^b^	36.4 ± 3.25^a^
UDC	0.10 ± 0.16^d^	5.50 ± 0.72^c^	12.8 ± 1.10^b^	25.5 ± 2.32^a^
B	0.00 ± 0.00^d^	5.00 ± 0.66^c^	10.3 ± 0.95^b^	17.8 ± 1.30^a^

Group I: Control, Group II: 250 µM MnCl_2_, Group III: 500 µM MnCl_2_, Group IV: 1000 µM MnCl_2_. Data are shown as mean ± SD (n = 10). MN and CAs were calculated by analyzing 1000 cells in each group, and MI by analyzing 10,000 cells in each group. The averages shown with different letters^(a–d)^ in the same line are statistically significant at p < 0.05. *MN* micronucleus, *FRG* fragment, *SC* sticky chromosome, *VC* vagrant chromosome, *UDC* unequal distribution of chromatin, *B* bridge.

These findings are similar to the results of a limited number of studies investigating Mn genotoxicity using plants as bioindicators. In literature, Kalefetoğlu Macar et al.^17^ investigated the toxicity of Mn exposure at doses of 100–500 µM using the *Allium* test. As a result, they reported a decrease in MI, an increase in MN frequency and an increase in CAs such as fragments, sticky chromosome, vagrant chromosome, unequal distribution of chromatin and bridge in *A. cepa* root tip cells, depending on the dose. Ertürk et al.^18^ used plant tissues as bioindicators and investigated Mn toxicity. As a result, they reported that the increase in Mn concentrations caused a decrease in the genomic stability and mitotic index of *Z. mays*, but an increase in DNA hypermethylation. Ghosh et al.^19^ determined that MnON particles at a dose of 20 μg/mL caused DNA hypomethylation in *Physcomitrium patens* gametophores known as algae. On the other hand, Chandra et al.^20^ used *A. cepa* test material to determine the genotoxicity of industrial wastewater containing metals such as manganese, and as a result, they determined that chromatid breaks and DNA fragmentation occurred in meristematic cells exposed to wastes containing MN. Obtaining similar results in this study with the literature studies investigating Mn toxicity using a bioindicator for the same purpose reveals the accuracy of the *Allium* test.

In this study, it is thought that the decrease in MI and the increase in the numbers of MN and CAs as a result of Mn exposure are thought to be caused by Mn interacting directly with DNA and microtubules or indirectly promoting damages through reactive oxygen species (ROS). Because detailed studies carried out in recent years have shown that heavy metals can produce ROS such as oxygen, carbon and sulfur radicals and the ability to bind directly to nucleic acids (DNA-heavy metal, protein-heavy metal cross-links). It has also been shown that both mechanisms can cause DNA damage such as single and double helix breaks, and oxidative degradation of nuclear proteins and DNA^21^. On the other hand, heavy metals also target microtubules that contribute to the organization of the most important cellular processes such as plant growth, development, mitotic spindle formation, cell plate, cell growth, elongation, intracellular transport and cell wall deposition. In this context, it has been reported that heavy metals reduce MI by causing deformation in the microtubule structure, disorder in the microtubule network, thickening of the microtubule fibers, and microtubule disassembly and de-polymerization^22^.

### Genotoxicity mechanism of MnCl_2_ confirmed by spectral shift

**Figure 4:**
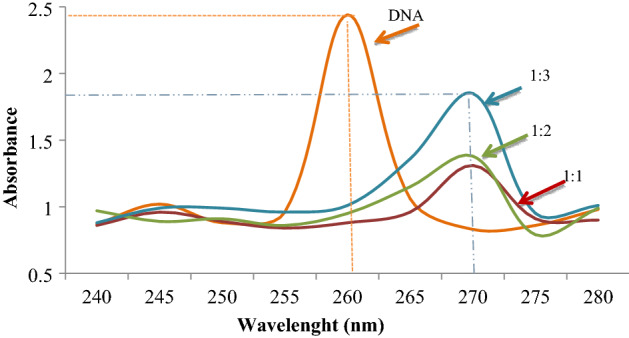
Spectral shift in UV spectrum of DNA after interaction with MnCl_2_. 1:1, 1:2, 1:3 show the DNA: MnCl_2_ ratios.

MnCl_2_-induced CAs and MN formations may result from the direct interaction of manganese with DNA, and to confirm this relationship, the spectral shift profile of DNA obtained from *Allium* root samples was investigated (Fig. 4). The standard UV–Vis spectrum of DNA shows a characteristic maximum peak at ∼ 260 nm, and a similar spectrum of DNA was obtained in this study. DNA–MnCl_2_ interaction caused bathochromic and hypochromic shifts in the UV spectrum. With the bathochromic shift observed after interaction with MnCl_2_, the UV spectrum of DNA shifted from 260 nm to about 270 nm. As a result of hypochromic shift, DNA absorbance decreased from 2.44 to 1.85 for 1:4 DNA–MnCl_2_ mixture. The severity of hypochromic shift increased as the MnCl_2_ dose increased. The hypochromic shift indicates that molecules interact with DNA via intercalation binding mode^23,24^. The hypochromic shift of the DNA–MnCl_2_ complex, which causes a decrease in absorbance, indicates the intercalation property of MnCl_2_. In interaction through intercalation, stacking occurs between base pairs without the formation of covalent bonds between chemicals and DNA. While this behavior indicates that intercalators are not DNA adductors, many chemicals exhibit promutagenic effects through intercalation. Intercalation can cause uncoiling of super-stranded DNA, inhibition of DNA and RNA synthesis, protein-associated DNA breaks and frameshift mutations^25,26^. The interaction of metal ions with DNA induces DNA compactisation^27^. Compacted DNA is more susceptible to breakage and especially to the toxic effects of chemicals. High CAs and MN formations resulting from DNA–MnCl_2_ interaction indicate genotoxic effect. The genotoxicity mechanism can be explained by the intercalation effect of MnCl_2_ detected by spectral shift.

### Biochemical observations

**Figure 5:**
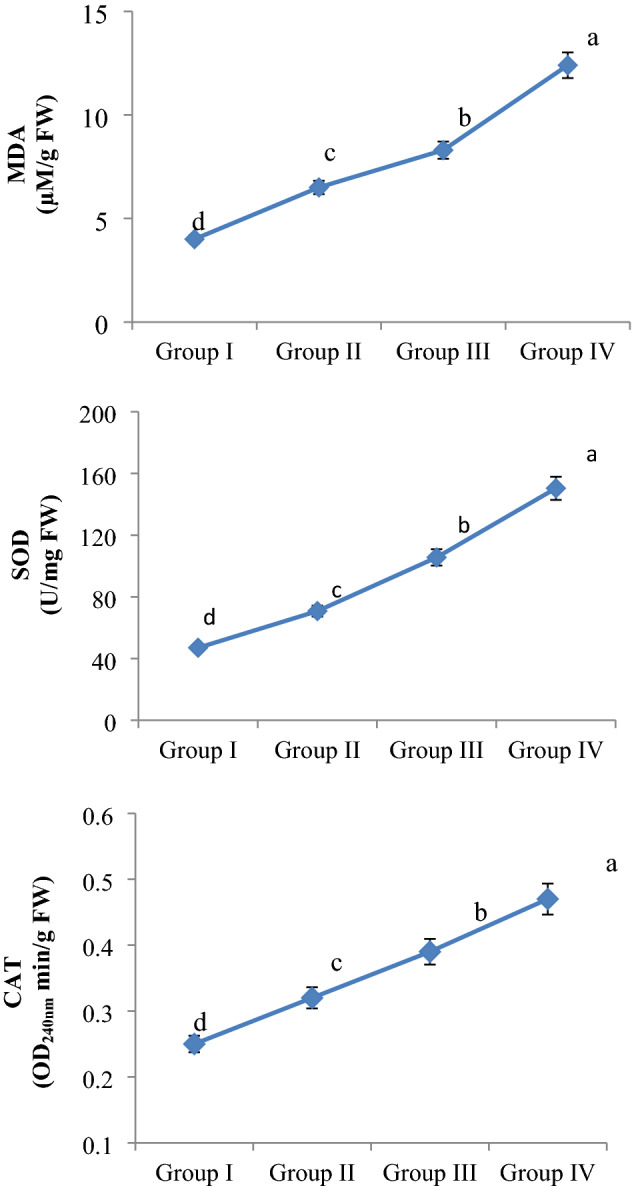
MnCl_2_ effects on MDA level, SOD and CAT enzyme activities. Group I: Control, Group II: 250 µM MnCl_2_, Group III: 500 µM MnCl_2_, Group IV: 1000 µM MnCl_2_. Data are shown as mean ± SD (n = 10). The averages shown with different letters^(a–d)^ are statistically significant at p < 0.05.

The effect of MnCl_2_ exposure on the biochemical parameters examined is shown in Fig. 5. The lowest MDA level and SOD and CAT enzyme activity were measured in the control group (group I). In this group, an average of 4.00 μM/g FW MDA levels, an average of 47.0 U/mg FW SOD enzyme activity and an average of 0.25 OD_240nm_ min/g CAT enzyme activity were measured, respectively. MnCl_2_ exposure caused statistically significant (p < 0.05) increases in MDA levels and SOD and CAT enzyme activities in all treatment groups, depending on the dose. It was determined that these increases were even more pronounced at 1000 µM dose of MnCl_2_. Compared to the control group, MDA level increased approximately 3.1 times, SOD activity increased approximately 3.2 times and CAT activity increased approximately 1.88 times in Group IV.

These results are very similar to the results of studies investigating the biochemical changes caused by excessive MnCl_2_ exposure in different plant species. For example, Sieprawska et al.^28^ reported that administration of MnCl_2_ at 1, 2 and 3 mM doses caused oxidative stress by promoting dose-dependent increases in MDA levels and decreases in SOD and CAT enzyme activities in wheat cells. Silva et al.^29^ determined that excessive MnCl_2_ exposure at a dose of 500 µM in *Zea mays* L. cv. *saracura* species caused an increase in lipid peroxidation (MDA) levels and antioxidant enzyme (SOD and CAT) activities in 7, 14 and 21-day periods. Xiao et al.^30^ determined that 1000, 5000, 10,000, 15,000 and 20,000 µM doses of MnCl_2_ increased MDA levels in the *Cleome viscosa* L. species known as Asian spider pox, while it caused an increase in SOD and CAT antioxidant enzyme activities up to 1000 µM doses and a decrease in other doses.

MDA is the main metabolite of arachidonic acid and is used as a reliable bio-indicator for oxidative stress. MDA is a mutagenic and highly reactive three-carbon di-aldehyde produced especially during polyunsaturated fatty acid peroxidation or arachidonic acid metabolism. MDA has the ability to covalently bind to proteins, RNA, and DNA. MDA also causes cross-linking in lipids. MDA is accepted as an important indicator of lipid peroxidation caused by especially free radicals in the cell membrane^31^. In this study, it is thought that the main reason for the increase in MDA levels in root cells as a result of MnCl_2_ exposure is that Mn accelerates lipid peroxidation by damaging the membranes of root tip meristem cells (directly or through the free radicals it creates). Because in recent studies, it has been reported that heavy metals disrupt the structure of cell membranes in plants and cause unsaturated fatty acids to turn into small hydrocarbon fragments such as MDA^32^.

SOD is the most powerful antioxidant enzyme in the cell. It takes part in the first-line defense system developed against ROS. It catalyzes the conversion of superoxide anion to H_2_O_2_ and molecular oxygen. As a result, the highly harmful superoxide anion is rendered relatively less harmful. CAT is a common tetrameric antioxidant enzyme found in almost all living things exposed to oxygen. It catalyzes the reduction of H_2_O_2_ to H_2_O and molecular oxygen. So much so that CAT can break down millions of H_2_O_2_ molecules in a second. Ultimately, it ensures the completion of the detoxification process initiated by SOD. SOD and CAT are the most important enzymes of the antioxidant system of organisms^33^. In this study, the reason for the increase in SOD and CAT enzyme activities in root tip cells as a result of MnCl_2_ exposure may be that Mn promotes free radical production in the cell and cells may increase SOD and CAT enzyme levels to reduce the harmful effects of free radicals produced. Because, Silva et al.^29^ reported that excessive Mn exposure induced an increase in ROS and H_2_O_2_ in plant cells, and that cells increases SOD and CAT activities as a protective mechanism against the oxidative damage created by these increased free radicals.

### Anatomical observations

**Figure 6:**
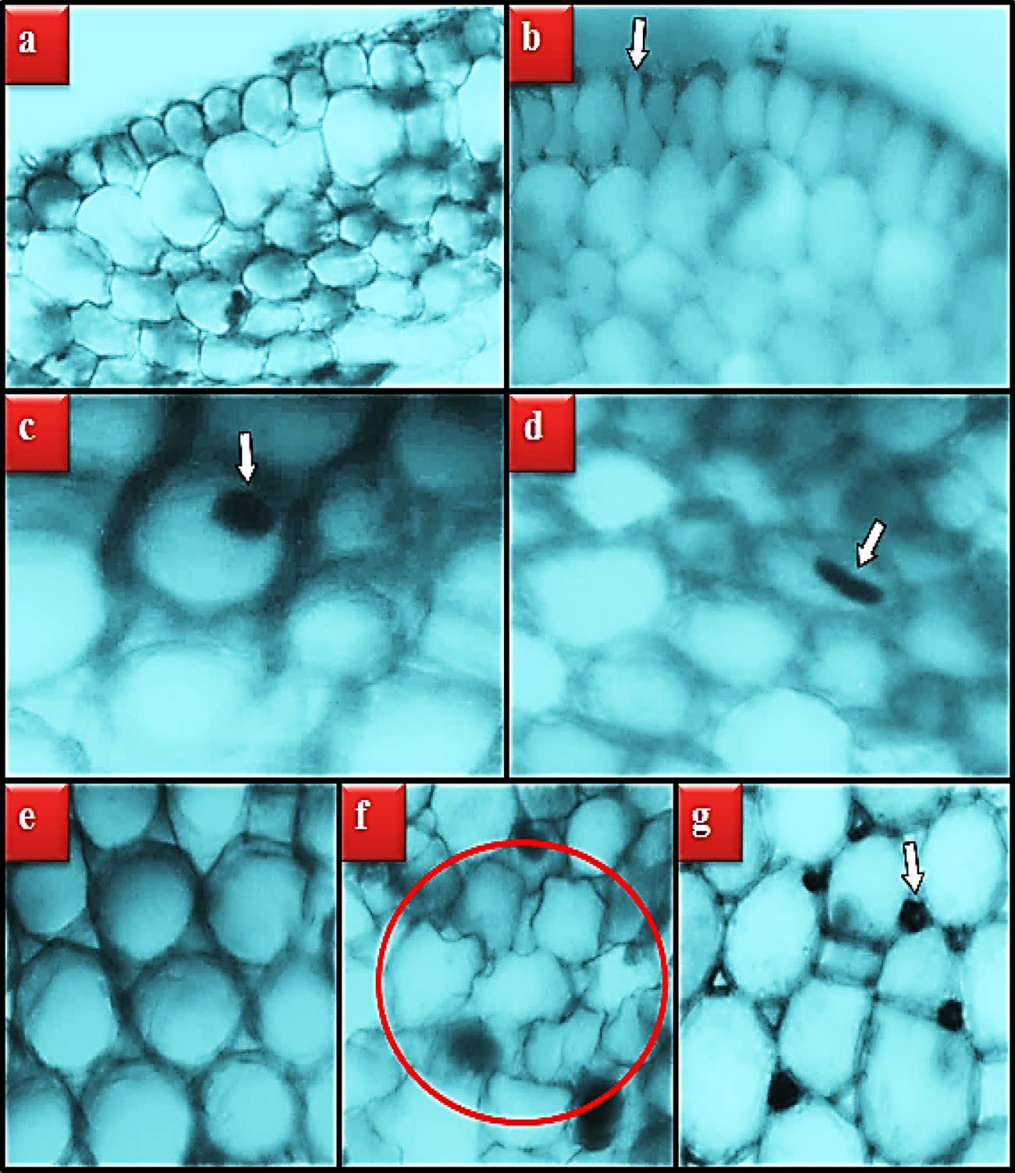
Meristematic cell damages caused by MnCl_2_ toxicity. Normal appearance of epidermis cells (**a**), epidermis cell damage (**b**), normal appearance of cell nucleus-*oval* (**c**), flattened cell nucleus (**d**), normal appearance of cortex cells (**e**), cortex cell damage-*red region*, cortex cell wall thickening (**g**).

The anatomical damages induced by MnCl_2_ exposure in the root tip meristematic cells of *A. cepa* are shown in Table 3 and Fig. 6. No damage was observed under the microscope in the root cells of the control group (group I). In the root tip cells of the groups exposed to MnCl_2_, damages such as epidermis cell damage, flattened cell nucleus, cortex cell damage and thickening of the cortex cell wall were observed in varying degrees depending on the dose.

**Table 3:** Effect of MnCl_2_ toxicity on root meristem cells.

Groups	ECD	FCN	CCD	CCWT
Group I	**–**	**–**	**–**	**–**
Group II	**+**	**+**	**+**	**+**
Group III	**++**	**++**	**++**	**+**
Group IV	**+++**	**+++**	**+++**	**++**

Group I: Control, Group II: 250 µM MnCl_2_, Group III: 500 µM MnCl_2_, Group IV: 1000 µM MnCl_2_. *ECD* epidermis cell damage, *FCN* flattened cell nucleus, *CCD* cortex cell damage, *CCWT* cortex cell wall thickening (–): no damage, (+): little damage, (++): moderate damage, (+++): severe damage.

Although there is no comprehensive study in the literature examining the anatomical changes caused by excessive MnCl_2_ exposure in plant cells, there are some studies conducted with other trace elements with heavy metal properties such as MnCl_2_. For example, Güler et al.^34^ reported that Cr application at doses of 2.4, 8.0, and 12.5 mg/L promoted anatomical damages such as cell deformation, thickening of the cortex cell wall, flattened cell nucleus, and necrosis in *A. cepa* root tip cells. Macar et al.^35^ found epidermis cell deformation, thickening of the cortex cell wall, and flattening of the cell nucleus in *A. cepa* root cells exposed to 20 µM Cu. Kalefetoğlu Macar et al.^36^ observed that Co exposure at a dose of 5.5 ppm caused deformation, thickening of the cell wall and flattening in the cell nucleus in *A. cepa* root cells.

Our study suggests that most of the anatomical damages observed in root cells as a result of MnCl_2_ exposure are caused by mechanical stress caused by the physical defense mechanisms developed by the cells to prevent Mn from being taken into the cell. In the examinations performed under the microscope, an increase in the number of epidermis and cortex cells was observed in the MnCl_2_ applied groups compared to the control group. These numerical increases developed by the cells to prevent Mn from entering the cell increase the contact of the cells with each other. Therefore, mechanical stress increases and this can cause deformities in epithelial and cortex cells and their nucleus. The information in the literature that plants have developed some chemical (increased synthesis of phytochelatin, metallothioneine and proline) and physical (thickening of the cell wall with an increase in cell number and cell layer) defense mechanisms to protect against heavy metal toxicity supports our idea^37,38^.

## Conclusion

Mn element is in the structure of the nutrients we consume abundantly in our daily life, the industrial products we frequently use and the fertilizers used in agricultural activities. It is an element that must be taken in trace amounts for the continuation of the metabolic activities of all organisms. However, it has been shown in *A. cepa*, that MnCl_2_ can cause physiological, cytogenetic, biochemical and anatomical toxicity in case of excessive intake. It has also been understood that the *Allium* test is a reliable test in determining this versatile toxicity. For this reason, avoiding excessive use of fertilizers containing Mn, especially in agricultural activities, and keeping the soil Mn amounts at an optimum level by regularly measuring them at regular intervals should be the primary priorities in preventing Mn toxicity.

## Data Availability

The datasets used and/or analyzed during the current study are available from the corresponding author on reasonable request.
